# Roles for T/B lymphocytes and ILC2s in experimental chronic obstructive pulmonary disease

**DOI:** 10.1002/JLB.3AB0518-178R

**Published:** 2018-09-27

**Authors:** Chantal Donovan, Malcolm R. Starkey, Richard Y. Kim, Batika M. J. Rana, Jillian L. Barlow, Bernadette Jones, Tatt Jhong Haw, Prema Mono Nair, Kurtis Budden, Guy J.M. Cameron, Jay C. Horvat, Peter A. Wark, Paul S. Foster, Andrew N. J. McKenzie, Philip M. Hansbro

**Affiliations:** ^1^ Priority Research Centres for Healthy Lungs and GrowUpWell Hunter Medical Research Institute and The University of Newcastle Newcastle New South Wales Australia; ^2^ Medical Research Council (MRC) Laboratory of Molecular Biology Cambridge UK; ^3^ The Centenary Institute and the School of Life Sciences University of Technology Sydney Sydney New South Wales Australia

**Keywords:** COPD, emphysema, ILC2s, inflammation, remodelling, T cells

## Abstract

Pulmonary inflammation in chronic obstructive pulmonary disease (COPD) is characterized by both innate and adaptive immune responses; however, their specific roles in the pathogenesis of COPD are unclear. Therefore, we investigated the roles of T and B lymphocytes and group 2 innate lymphoid cells (ILC2s) in airway inflammation and remodelling, and lung function in an experimental model of COPD using mice that specifically lack these cells (*Rag1^−/−^* and *Rora^fl/fl^Il7r* ^Cre^ [ILC2‐deficient] mice). Wild‐type (WT) C57BL/6 mice, *Rag1* ^−/−^, and *Rora^fl/fl^Il7r* ^Cre^ mice were exposed to cigarette smoke (CS; 12 cigarettes twice a day, 5 days a week) for up to 12 weeks, and airway inflammation, airway remodelling (collagen deposition and alveolar enlargement), and lung function were assessed. WT, *Rag1* ^−/−^, and ILC2‐deficient mice exposed to CS had similar levels of airway inflammation and impaired lung function. CS exposure increased small airway collagen deposition in WT mice. *Rag1* ^−/−^ normal air‐ and CS‐exposed mice had significantly increased collagen deposition compared to similarly exposed WT mice, which was associated with increases in IL‐33, IL‐13, and ILC2 numbers. CS‐exposed *Rora^fl/fl^Il7r* ^Cre^ mice were protected from emphysema, but had increased IL‐33/IL‐13 expression and collagen deposition compared to WT CS‐exposed mice. T/B lymphocytes and ILC2s play roles in airway collagen deposition/fibrosis, but not inflammation, in experimental COPD.

## 1 INTRODUCTION

Chronic obstructive pulmonary disease (COPD) is the third leading cause of death globally and is characterized by progressive airway inflammation, emphysema, and reduced lung function.^1^, ^2^, ^3^, ^4^ Chronic pulmonary inflammation in COPD is underpinned by innate immune responses and primarily involves macrophages (alveolar and interstital), neutrophils, dendritic cells, natural killer cells, and innate lymphoid cells (ILCs).^5^ ^–7^ This is associated with concomitant increases in the number of adaptive immune cells, primarily infiltrating CD4^+^ and CD8^+^ T and B lymphocytes.^5^, ^6^

Although limited in number in the lung, ILCs are crucial in bridging innate and adaptive immune responses and in maintaining lung homeostasis.^8^, ^9^ They are characterized into three subsets (ILC1, ILC2, ILC3) and are tissue resident lymphocytes that may display considerable plasticity depending on the stimulus.^10^, ^11^ In COPD, ILC2s phenotypically shift into ILC1 and ILC3 subsets upon stimulation with IL‐1β, IL‐4, and IL‐12^10^ and the increased ratio of ILC1:ILC2 directly correlates with a decline in lung function and more severe disease.^11^ In mouse models of COPD, cigarette smoke (CS) exposure has similar suppressive effects on ILC2s.^12^ Although the endogenous stimulus that switches ILC subsets and their role in exacerbations of COPD has been studied to some degree,^11^, ^12^ the role of ILC2s in the pathogenesis of COPD is poorly understood, particularly their role in airway remodelling and lung function decline.

Along with innate cells, there is an increase in adaptive immune cells, predominatory CD4^+^ and CD8^+^ T lymphocytes and B lymphocytes in both the large and peripheral airways in patients with COPD.^13^, ^14^, ^15^, ^16^ In mice exposed to CS, CD8‐deficient (^−/−^), but not CD4^−/−^ mice, are protected from CS‐induced hallmark features of COPD, specifically macrophage accumulation, MMP2 and MMP9 activity, and emphysema,^17^, ^18^ indicating a key role of CD8^+^ lymphocytes in disease pathogenesis. In addition, B cells, in particular B cell rich lymphoid follicles, are increased in the severe stages of COPD and are most strongly linked with the emphysematous changes in the lung (reviewed in Ref. 19). Autoantibodies are also evident in the blood and lung samples from COPD patients.^20^, ^21^, ^22^ B cell‐activating factor (BAFF) is significantly increased around immune cells and within lymphoid follicles in COPD patients, and blocking BAFF in a CS‐induced mouse model of COPD reduces inflammation and alveolar destruction.^23^ Interestingly, mice lacking mature T and B lymphocytes (*Rag2^−/−^*), had no change in inflammation and emphysema compared to wild‐type (WT) controls.^24^ However, these mice developed COPD‐like disease when CD3^+^ T‐cells from CS‐exposed WT mice were adoptively transferred,^24^ demonstrating a key role of pathogenic T cells in the development of COPD. Crucially, the roles of T and B cell deficiency on airway remodelling (collagen deposition) and lung function in experimental COPD have not been assessed.

In this study, we further elucidated the roles of T/B lymphocytes and ILC2s in CS‐induced experimental COPD. We first used an established model of experimental COPD^25^, ^26^, ^27^, ^28^, ^29^, ^30^, ^31^, ^32^ and WT mice to define subsets of innate lymphoid cells, and ILC2s in *Rag1* ^−/−^,^33^ and then assessed CS‐induced changes in ILC2‐deficient mice (*Rora^fl/fl^Il7r* ^Cre^). WT, *Rag1* ^−/−^, and ILC2‐deficient mice had similar levels of airway inflammation following CS exposure. However, *Rag1^−/−^* mice developed spontaneous increases in collagen deposition in the presence or absence of CS, which was associated with increased IL‐33, IL‐13, and ILC2 numbers. ILC2‐deficient mice were protected from CS‐induced emphysema, but had increased collagen deposition, and IL‐33 and IL‐13 expression. Collectively, these data show roles for T and B lymphocytes and ILC2s in CS‐induced airway remodelling and emphysema, but not inflammation, in experimental COPD.

## 2 METHODS

### 2.1 Mice

All procedures were approved by the University of Newcastle Animal Care and Ethics Committee.

### 2.2 Cigarette smoke exposure

WT C57BL/6, *Rag1* ^−/−^,^33^ *Rora^fl/+^Il7r* ^Cre^ (heterozygous controls), and *Rora^fl/fl^Il7r* ^Cre^ (ILC2‐deficient) mice^34^ were exposed to the smoke of twelve 3R4F reference cigarettes (University of Kentucky, Lexington, KY) twice per day, 5 times a week for up to 12 weeks, as previously described.^25^, ^26^, ^27^, ^28^, ^29^, ^30^, ^31^, ^32^

### 2.3 Airway inflammation, airway remodelling, and emphysema

Airway inflammation and remodelling as well as emphysema were measured, as previously described.^25^, ^26^, ^27^, ^28^, ^29^, ^30^, ^31^, ^32^ Bronchoalveolar lavage fluid (BALF) was obtained by 2 × 0.5 ml PBS washes of the left lobe only *via* a cannula inserted into the trachea. Airway inflammation was measured by differential enumeration of inflammatory cells with May‐Grunswald staining, as previously described.^25^, ^26^, ^28^ The left lobe was then perfused *via* the heart and inflated with, and drop‐fixed in formalin, prior to paraffin embedding and sectioning (4 μm thick). Masson's trichrome staining was used to measure collagen deposition and H&E staining for assessment of emphysema‐like alveolar enlargement, as previously described.^25^, ^26^

### 2.4 Qpcr

Lungs were homogenized and total RNA was isolated using TRIzol Reagent (Invitrogen, Life Technologies, Australia). Random‐primed reverse transcriptions were performed using BioScript reverse transcriptase in one times first‐strand buffer according to the manufacturer's instructions (Bioline Pty. Ltd., NSW, Australia). Real‐time qPCR assays were performed with SYBR Green Supermix (KAPA Biosystems, Inc., MA) and a Mastercycler ep realplex2 system (Eppendorf South Pacific Pty. Ltd., NSW, Australia). IL‐33 and IL‐13 gene expression was normalized to the housekeeping gene hypoxanthine guanine phosphoribosyltransferase (*Hprt*). *Hprt*: forward 5′‐AGGCCAGACTTTGTTGGATTTGAA‐3′; *Hprt*: reverse 5′‐CAACTTGCGCTCATCTTAGGCTTT‐3′; *Il 33*: forward 5′‐CCTCCCTGAGTACATACAATGACC‐3′; *Il 33*: reverse: 5′‐GTAGTAGCACCTGGTCTTGCTCTT‐3′; *Il 13*: forward: 5′‐TGCTTGCCTTGGTGGTCT‐3′; and *Il 13*: reverse: 5′‐GGGGAGTCTGGTCTTGTGTG‐3′.

### 2.5 ELISA

IL‐33 concentrations in whole lung homogenates were determined using a duoset ELISA kit (R&D Systems, InVitro Technologies, Noble Park, NSW, Australia) according to the manufacturer's instructions.^35^

### 2.6 Flow cytometry

Whole lung single cell suspensions were prepared, as previously described,^35^, ^36^ and incubated with Fc block (10 ng/ml, BD Biosciences, North Ryde, NSW, Australia; in 2% fetal calf serum in PBS). Cell surface antigens were labelled for the identification of inflammatory cells (ILC1s, ILC2s, ILC3s; Tables 1 and 2). Cells were analyzed on a BD FACSAriaIII and resulting data analyzed using FlowJo software.

**Table 1:** Flow cytometry panel for ILC2s

Antigen	Flurochrome	Company	Clone
CD45	PerCP‐Cy5.5	Biolegend	30‐F11
Lineage	AF700	Biolegend	
CD90.2	APC‐Cy7	Biolegend	30‐H12
IL‐7Rα	BV605	Biolegend	A7R34
CD25	APC	Biolegend	PC61
CD2	FITC	Biolegend	RM2‐5
Rorγt	PE	Biolegend	Q31‐378
GATA3	PE‐Cy7	BD	L50‐823
T‐bet	BV421	BD	04‐46

**Table 2:** Flow cytometry panel for ILCs

Antigen	Fluorochrome	Company	Clone
ILC1, 2, 3 with transcription factors			
Lineage	AF700	Biolegend	
CD45	PerCP‐Cy5.5	Biolegend	30‐F11
CD90.2	APC‐Cy7	Biolegend	30‐H12
IL‐7Rα	BV605	Biolegend	A7R34
Rorγt	PE	Biolegend	Q31‐378
GATA3	PE‐Cy7	BD	L50‐823
T‐bet	BV421	BD	04‐46
CD4	FITC	Biolegend	GK1.5

ILC1: Lin^‐^CD90.2^+^CD45^+^IL‐7Rα^+^T‐bet^+^; ILC2: Lin^‐^CD90.2^+^CD45^+^IL‐7Rα^+^GATA3^+^T‐bet^‐^Rorγt^‐^CD4^−^; ILC3: Lin^‐^CD90.2^+^CD45^+^IL‐7Rα^+^Rorγt^+^CD4^+/−^

### 2.7 Lung function

Mice were anaesthetized with ketamine/xylazine prior to lung function analysis using forced oscillation techniques and restrained plethysmography with methacholine (MCh) provocation, as previously described.^25^, ^28^, ^37^, ^38^, ^39^, ^40^, ^41^

### 2.8 RT2 extracellular matrix (ECM) PCR array

RT2 ECM and adhesion molecule array (Qiagen, Chadstone Centre, VIC, Australia) was performed according to the manufacturer's instructions.

### 2.9 Statistical analyses

Data are expressed as mean ± sem. Nonnormally distributed data were analyzed using nonparametric equivalents. Comparisons between two groups were made using a two‐tailed Mann‐Whitney test. Multiple comparisons were made using one‐way ANOVA with Tukey's posttest, or the Kruskal‐Wallis test with multiple comparisons for nonparametric analyses. *P* < 0.05 was considered statistically significant.

## 3 RESULTS AND DISCUSSION

### 3.1 Airway remodelling and resistance are increased in association with elevated baseline IL‐33, IL‐13, and ILC2 numbers, but pulmonary inflammation and emphysema‐like alveolar enlargement are unaltered in *Rag1^−/−^* mice compared to WT mice with experimental COPD

**Figure 1:**
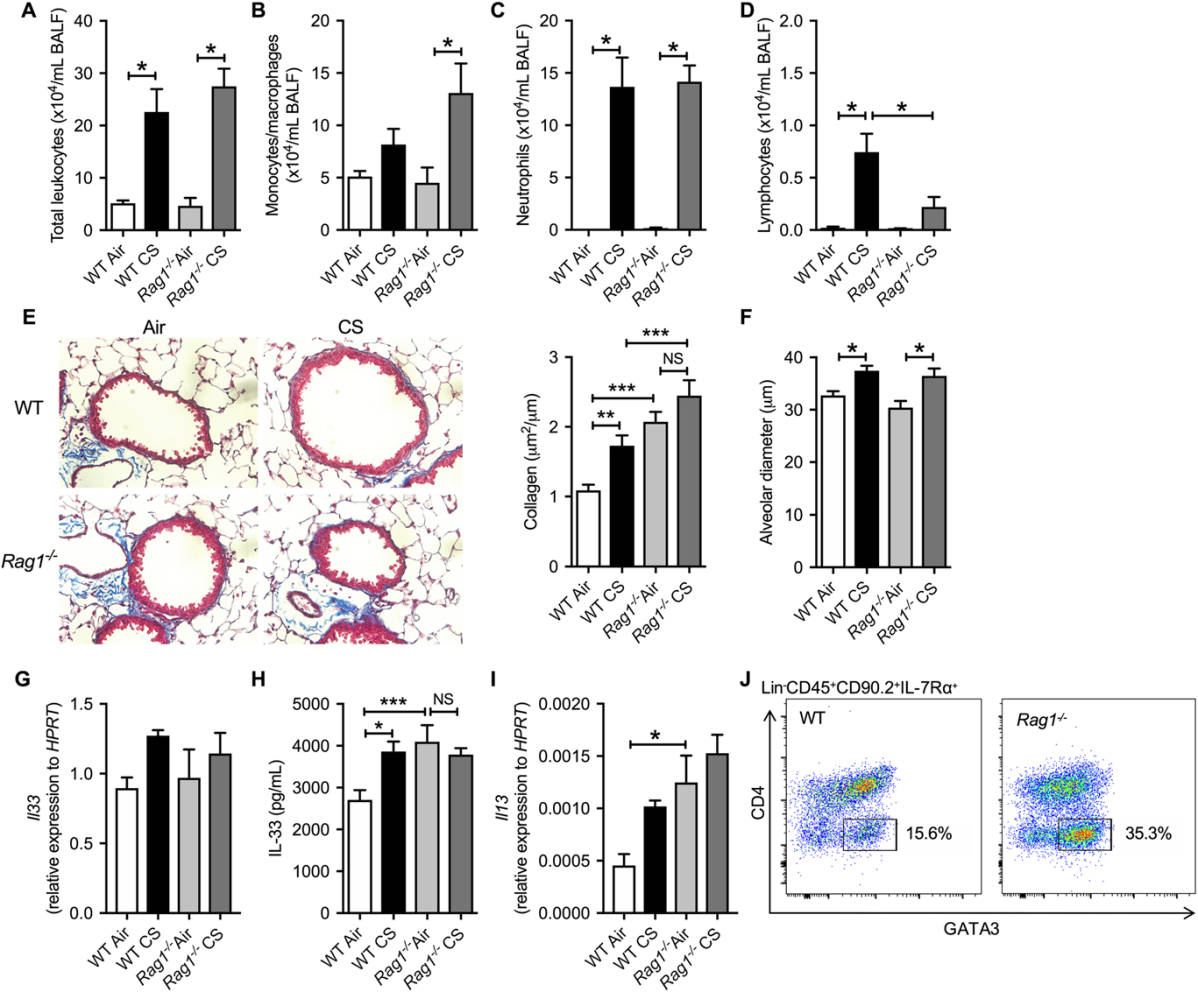
**Airway remodelling is increased in association with elevated baseline IL‐33, IL‐13, and ILC2s, but inflammation and emphysema‐like alveolar enlargement are largely unaltered in CS‐exposed *Rag1^−^^/^^−^* compared to WT mice**. Mice were lavaged and (A) total BAL cells, (B) monocytes/macrophages, (C) neutrophils, and (D) lymphocytes were enumerated. (E) Representative images and quantification of collagen, and (F) quantification of alveolar diameter in normal air‐ or CS‐exposed WT and *Rag1^−/−^* mice. Whole lung (G) Il33 mRNA expression, (H) IL‐33 protein levels and (I) Il13 mRNA expression. (J) Flow cytometric analysis of ILC2s in normal air‐exposed WT versus *Rag1^−/−^* mice. *n* = 4–6 per group. Data are expressed as mean ± sem. *P < 0.05, **P < 0.01, ***P < 0.001

Mice were exposed to CS for 8 weeks to induce experimental COPD, as we have previously shown.^25^, ^26^, ^27^, ^28^, ^29^, ^30^, ^31^, ^32^ CS exposure impaired weight gain (data not shown) and increased total leukocyte numbers (comprised primarily of neutrophils and lymphocytes) in the airways in WT mice, as previously described (Fig. 1A‐D).^25^, ^29^, ^31^ Similar observations were made with *Rag1^−/−^* mice. The only exception was that CS‐exposed *Rag1^−/−^* mice had reduced numbers of lymphocytes in the BALF compared to CS‐exposed WT mice (Fig. 1D). This is consistent with previous reports in *Rag2^−/−^* mice due to a lack of B and T cells.^24^ The differences between *Rag1* and *Rag2* relate to their binding to the V(D)J recombination. Depleting either *Rag1* or *Rag2* has the same effect, which is defective expression of pre‐TCR and pre‐BCR, meaning that *Rag1^−/−^* or *Rag2^−/−^* mice cannot generate mature T and B cells.^42^ The remaining lymphocytes in CS‐exposed *Rag1^−/−^* mice could potentially be ILCs; however, further flow cytometric analysis of these cells is required. We next assessed the effects of CS exposure on pulmonary remodelling and emphysema‐like alveolar enlargement, and lung function in *Rag1^−/−^* mice. CS exposure increased collagen deposition around the small airways in WT mice (Fig. 1E). Collagen deposition was increased around the small airways in CS‐exposed *Rag1^−/−^* compared to WT controls. *Rag1^−/−^* mice exposed to normal air had spontaneous collagen deposition around some airways, which was intermediate between levels in CS‐exposed WT and *Rag1^−/−^* groups (Fig. 1E). CS exposure resulted in similar levels of alveolar enlargement in WT and *Rag1^−/−^* mice (Fig. 1F). Airway resistance was increased in *Rag1^−/−^* CS‐exposed mice compared to *Rag1^−/−^* air‐exposed mice (0.17 ± 0.09 *cf*. 0.09 ± 0.02 cmH_2_O.s/mL); however, there was no significant difference compared to WT air and CS‐exposed mice (data not shown). The disconnect between increased collagen deposition and lung function needs further in‐depth study.

We next assessed factors and cell types that are known to play roles in airway remodelling, namely, IL‐33, IL‐13, and ILC2s.^43^, ^44^ In WT mice, CS exposure increased lung IL 33 mRNA and protein, and IL‐13 mRNA expression compared to normal air‐exposed controls (Fig. 1G‐I). Conversely, there were no increases observed in CS‐exposed *Rag1^−/−^* mice compared to their normal air‐exposed controls; however, this was largely due to increased baseline levels of IL‐33 and IL‐13 in the *Rag1^−/−^* control mice (Fig. 1H, I). Flow cytometric analysis revealed increases in Lin^−^CD45^+^CD90.2^+^IL‐7Rα^+^GATA3^+^ ILC2s in *Rag1^−/−^* compared to WT mice at baseline (i.e., without any challenge; Fig. 1J). It is established that IL‐33 can induce the proliferation of ILC2s that leads to increased IL‐13 production and profibrogenic cytokine production resulting in collagen deposition.^43^ Our observations that IL‐33 protein, IL‐13 mRNA, and ILC2 numbers were all increased in normal air‐exposed *Rag1^−/−^* mice suggest that the deficiency in T/B lymphocytes increases innate immune responses involving ILC2s. In CS‐exposed *Rag1^−/−^* mice, there were no further increases in collagen deposition, IL‐33 or IL‐13 compared to their normal air‐exposed controls. Nevertheless, CS‐exposed *Rag1^−/−^* mice had increased collagen deposition and trends toward increased IL‐13 mRNA levels, but not alveolar enlargement, compared to CS‐exposed WT mice. Our results are consistent with other studies that used *Rag2^−/−^* mice, which showed that they are not protected from CS‐induced alveolar enlargement.^17^, ^18^ There is little evidence that CD8^+^‐deficient mice may be protected,^24^ suggesting a yet to be identified mechanism for the lack of protection in T and B cell deficient mice. In addition, *MMP8* mRNA was further increased in *Rag1^−/−^* CS‐exposed mice compared to WT CS, and *Tgfbi* and *Timp1* transcript levels were increased in normal air and further increased in CS‐exposed *Rag1^−/−^* mice (Supplementary Fig. 1A). MMP8, TGF‐β‐induced, and Timp1 have all been shown to contribute to other fibrotic diseases^45^, ^46^, ^47^; however, their specific role(s) in COPD pathogenesis are unclear. Taken together, these data show that T/B lymphocyte deficiency increases IL‐33/IL‐13/ILC2 responses and *MMP8, Tgfbi*, and *Timp1*, which may be associated with increased collagen deposition/fibrosis, but not emphysema‐like changes in experimental COPD.

### 3.2 Increased lung ILC1/ILC3 and reduced ILC2 numbers occur in WT mice with experimental COPD

Given that the absence of mature T and B lymphocytes in *Rag1^−/−^* mice did not protect against cardinal features of COPD, we profiled changes in ILCs following 8 weeks of CS exposure in WT mice. Similar to previously published human severe COPD patient data,^10^ we found that CS exposure did not alter total lung ILC numbers (Lin^−^CD90.2^+^CD45^+^IL‐7Rα^+^) in WT mice but increased the proportion of ILC1s and ILC3s and decreasing that of ILC2s (Supplementary Fig. 2A‐E). This is similar to human studies,^11^, ^12^ where the total numbers of ILCs in the lung were unaltered by CS exposure. T‐bet^+^ ILCs (ILC1s) were increased 6‐fold and Rorγt^+^ ILCs (ILC3s) increased >3‐fold following CS exposure; however, there was no change in IFN‐γ or IL‐17 protein levels in lung tissues (by ELISA, data not shown). Thus, the development of CS‐induced experimental COPD is associated with a phenotypic switch from ILC2s to ILC1s and ILC3s. It is known that CS exposure impairs lung Th2 responses, which is due to reduced ILC responsiveness to IL‐33, and compensatory increases in natural killer cell responses to IL‐33.^12^

### 3.3 Pulmonary inflammation is largely unaltered, airway neutrophil numbers are reduced, airway collagen, IL‐33 and IL‐13 expression are increased, and emphysema‐like alveolar enlargement is inhibited in CS‐exposed ILC2‐deficient mice

We then assessed the roles of ILC2s in experimental COPD using mice deficient in ILC2s (*Rora^fl/fl^Il7r^Cre^*). Normal air‐exposed ILC2‐deficient mice gained significant body weight over 12 weeks compared to their heterozygous and WT controls (data not shown). This is consistent with a previous report that dysfunctional ILC2s can lead to impaired beige adipocyte function and increase weight gain.^48^

**Figure 2:**
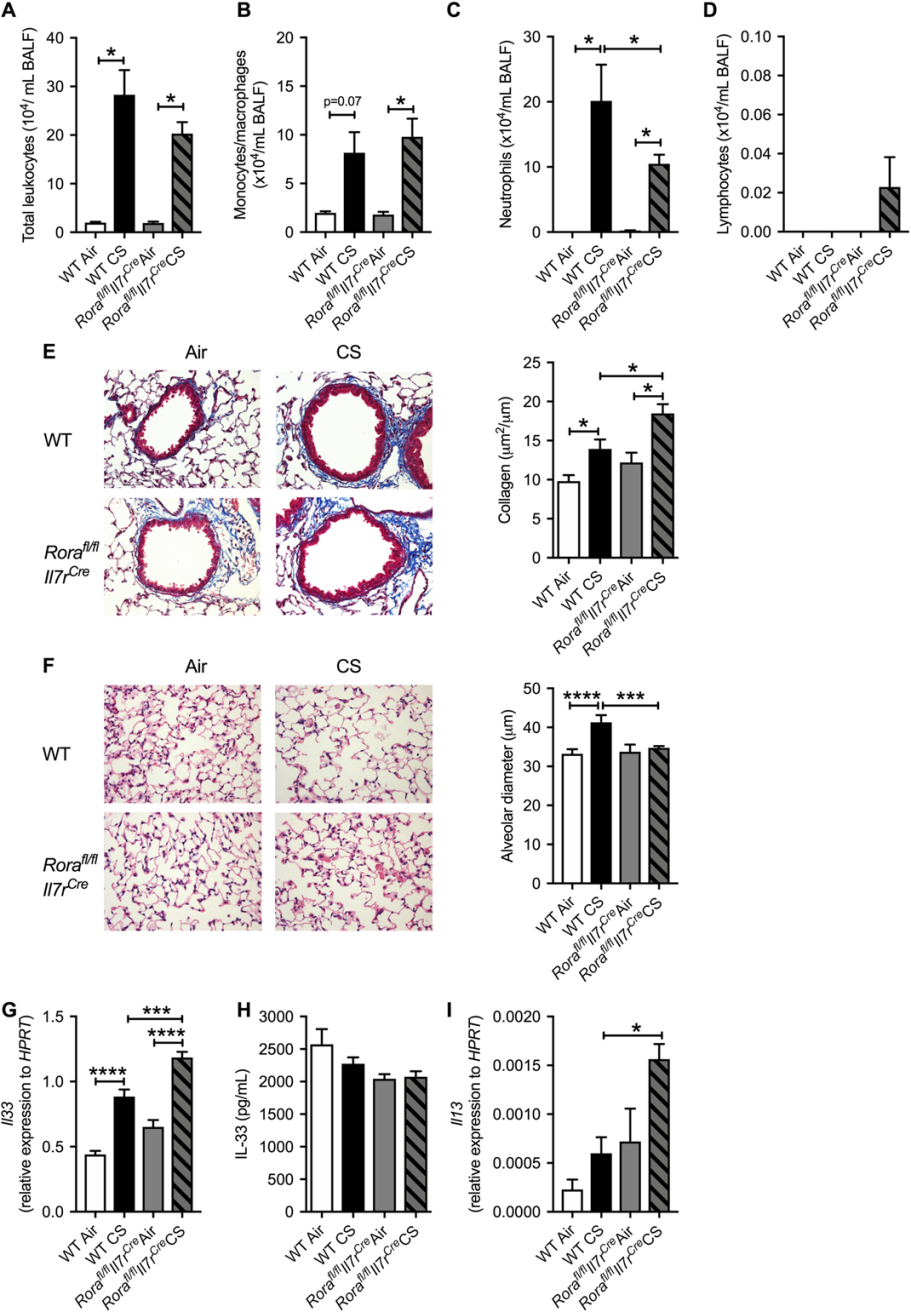
**Pulmonary inflammation is largely unaltered, airway neutrophil numbers are reduced, airway remodelling is increased in association with Il33 and Il13 mRNA expression, and emphysema‐like alveolar enlargement is inhibited in ILC2‐deficient mice exposed to CS**. Mice were lavaged and (A) total BAL cells, (B) monocytes/macrophages, (C) neutrophils, and (D) lymphocytes were enumerated. Representative images and quantification of (E) collagen and (F) alveolar diameter in normal air‐ or CS‐exposed WT and Rora^fl/fl^Il7r^Cre^ (ILC2‐deficient) mice. Whole lung (G) Il33 mRNA expression, (H) IL‐33 protein levels, and (I) Il13 mRNA expression. *n* = 4–8 per group. Data are expressed as mean ± sem. *P < 0.05, **P < 0.01, ***P < 0.001, ****P < 0.0001

Notably, when exposed to CS ILC2‐deficient mice had equivalent levels of impaired weight gain as CS‐exposed WT controls (data not shown). CS increased total leukocyte numbers in the airways, which was similar in WT and ILC2‐deficient mice (Fig. 2A). These cells were predominately macrophages (Fig. 2B) and neutrophils (Fig. 2C), with fewer neutrophils observed in CS‐exposed ILC2‐deficient compared to WT mice. Lymphocyte numbers were increased in CS‐exposed ILC2‐deficient mice (Fig. 2D). Similar weight and leukocyte numbers trajectories were observed in normal air or CS‐exposed *Rora^fl/+^Il7r^Cre^* heterozygous mice compared to WT controls (data not shown). Although the exact mechanisms driving the decrease in neutrophils are yet to be elucidated it is known that IL‐33/ILC2 activation can regulate neutrophil IL‐5 positivity following acute lung injury.^49^ Thus, mice lacking ILC2s may have impaired regulation and activation of neutrophils. Our study is the first to show that ILC2s play a role in CS‐induced neutrophilic airway inflammation.

We next assessed pulmonary remodelling in ILC2‐deficient mice. Again, there was increased collagen deposition around the small airways following CS exposure in WT mice (Fig. 2E). CS exposure also increased collagen levels around the small airways in ILC2‐deficient mice, with the level of deposition higher than that observed in CS‐exposed WT mice. CS exposure also induced alveolar enlargement in WT mice whereas ILC2‐deficient mice were completely protected against this major pathologic feature of COPD (Fig. 2F). These results are similar to elastase‐induced emphysematous lung pathology, whereby the absence of ILC‐intrinsic arginase‐1 prevented disease development.^8^ However, our results are in contrast to a study demonstrating that *Nippostrongylus brasiliensis* challenge in mice lacking IL‐9, which is crucial for ILC2 survival, increased emphysema compared to WT mice.^50^ There was no change in airway resistance at baseline (data not shown).

We next assessed the levels of lung IL‐33 and IL‐13.^43^, ^44^ CS exposure increased lung *Il 33* expression in WT mice, which was further increased in CS‐exposed ILC2‐deficient mice (Fig. 2G‐I); however, there were no differences in lung IL‐33 protein levels. *Il13* mRNA expression was significantly increased in CS‐exposed ILC2‐deficient mice compared to CS‐ and normal air‐exposed WT controls. These data suggest that CS‐induced increases in IL‐33, IL‐13, and collagen deposition are independent of ILC2s. Previous studies in asthmatics have shown that IL‐33 can increase IL‐13 through mast cell activation,^51^ and we previously demonstrated key roles for two mast cell tryptases in the pathogenesis of COPD.^25^, ^28^ In addition, *MMP8, Tgfbi*, and *Timp1* levels were increased in CS‐ compared to normal air‐exposed WT and ILC2‐deficient mice. However, there were no additional increases in CS‐exposed ILC2‐deficient compared to WT mice (Supplementary Fig. 1B). Together, our data suggest that in the absence of ILC2s, IL‐33 can induce IL‐13 responses and downstream pro‐fibrotic pathways to increase collagen deposition and emphysema, which may in part be regulated by *MMP8, TGFbi*, and *Timp1*. In light of previous reports, these effects may also involve mast cell responses.

### 3.4 Body weight increase in ILC2‐deficient mice associates with airway hyperresponsiveness

**Figure 3:**
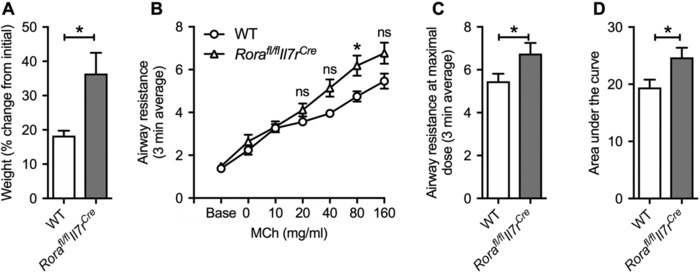
**Increased weight and airway hyperresponsiveness occurs in ILC2‐deficient exposed to normal air**. (A) Weights of normal air‐exposed WT and Rora^fl/fl^IL7r^Cre^ mice. (B) Airway resistance measured in response to increasing doses of methacholine (MCh). (C) Airway resistance at the maximal 160 mg/ml dose of MCh. (D) Area under the curve analysis of airway resistance. *n* = 4–23 per group. Data are expressed as mean ± sem. **P < 0.01

Normal air‐exposed ILC2‐deficient (*Rora^fl/fl^Il7r^Cre^*) mice gained more weight than WT mice (Fig. 3A). We next assessed whether normal air‐exposed ILC2‐deficient mice had altered lung function in response to airway provocation. ILC2‐deficient mice had airway hyperresponsiveness in response to MCh at 80 mg/ml or higher (Fig. 3B‐C), and increased resistance measured by area under the curve (Fig. 3D) compared to WT controls. These data demonstrate that impaired baseline lung function in normal air‐exposed ILC2‐deficient mice is also associated with increased airway hyperresponsiveness, which may be attributed to increased weight gain. Whereas the roles of ILC2s in other respiratory diseases such as asthma are well established, the impact of depleting ILC2s on lung function parameters has not been assessed. Increased mass loading on the chest wall in obese humans can alter the balance of forces within the respiratory system that result in increased airway resistance and decreased total lung capacity as we found in ILC2‐deficient mice.^52^ Thus, our data also highlight a potential role for ILC2s in maintaining lung homeostasis.^9^

This study demonstrates important roles for T/B lymphocytes and ILC2s in airway remodelling in COPD. It is already well known that there is a disproportionate increase in collagen and ECM deposition in COPD patients compared to asthmatics, and that this increase contributes to fixed airflow obstruction.^53^ Our data increases our understanding of the roles of T/B lymphocytes and ILC2s in the pathogenesis of experimental COPD, and highlights the potential for innate and adaptive immune cross‐talk in COPD.

## DISCLOSURES

The authors declare no conflicts of interest.

## Supporting information

Supplementary Fig.  1: Top 5 differentially expressed genes identified from RT2 ECM PCR array in WT, Rag1^−/−^ and Rora^fl/fl^IL7R^Cre^ mice exposed to CS. Relative abundance of the top 5 genes compared to the geometric mean of Actb, B2m, Gapdh, for normal air‐ and CS‐exposed A) Rag1^−/−^ mice and B) Rora^fl/fl^IL7R^Cre^ mice compared to WT controls. cDNA was pooled from 6 samples in each group.

Supplementary Fig. 2: T‐bet+ ILC1s and Rorγt+ ILC3s are increased and GATA3+ ILC2s are decreased in WT mice exposed to CS. Flow cytometric analysis of single cell suspensions from whole lungs of normal air‐ or CS‐exposed WT mice. (A) Flow cytometry gating of ILC1s, ILC2s and ILC3s. Quantification of (B) total ILCs, (C) ILC1s, (D) ILC2s, and (E) ILC3s. n = 5‐6 per group. Data are expressed as mean ± sem. * P < 0.05, ** P < 0.01. Panels: ILC1: Lin−CD90.2+CD45+IL‐7Rα+T‐bet+; ILC2: Lin−CD90.2+CD45+IL‐7Rα+GATA3+T‐bet−Rorγt−CD4−; ILC3: Lin−CD90.2+CD45+IL‐7Rα+Rorγt+CD4+/−.
