# Supplementary figures and images for: Roles for T/B lymphocytes and ILC2s in experimental chronic obstructive pulmonary disease
Source: J Leukoc Biol. 2018 Sep 27;105(1):143–50. doi: 10.1002/JLB.3AB0518-178R (PMC6487813; doi:10.1002/JLB.3AB0518-178R)

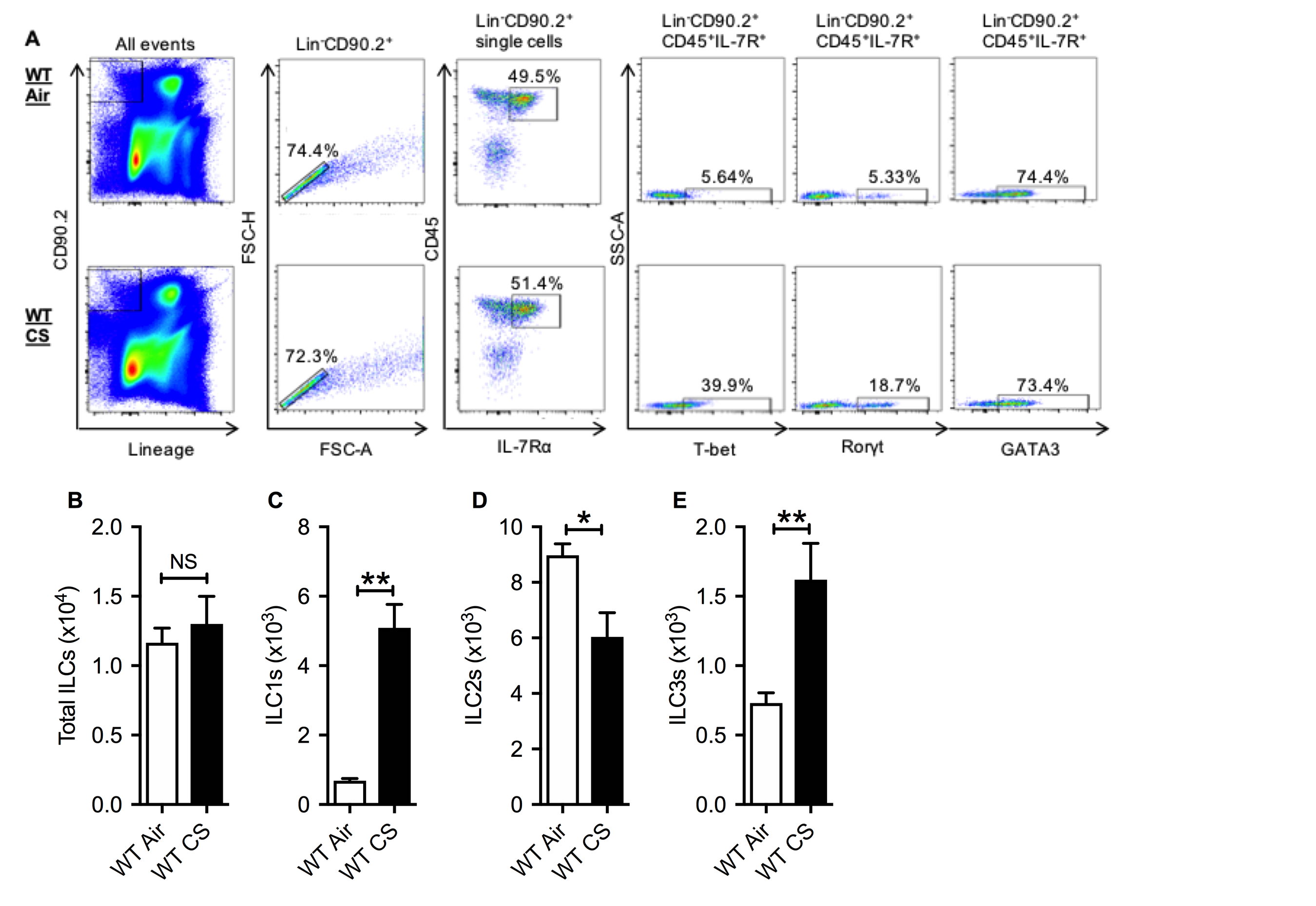

Supplement: Supplementary file 1 — Supplementary Fig. 1: Top 5 differentially expressed genes identified from RT2 ECM PCR array in WT, Rag1−/− and Rorafl/flIL7RCre mice exposed to CS. Relative abundance of the top 5 genes compared to the geometric mean of Actb, B2m, Gapdh, for normal air‐ and CS‐exposed A) Rag1−/− mice and B) Rorafl/flIL7RCre mice compared to WT controls. cDNA was pooled from 6 samples in each group. [file JLB-105-143-s001.tiff]

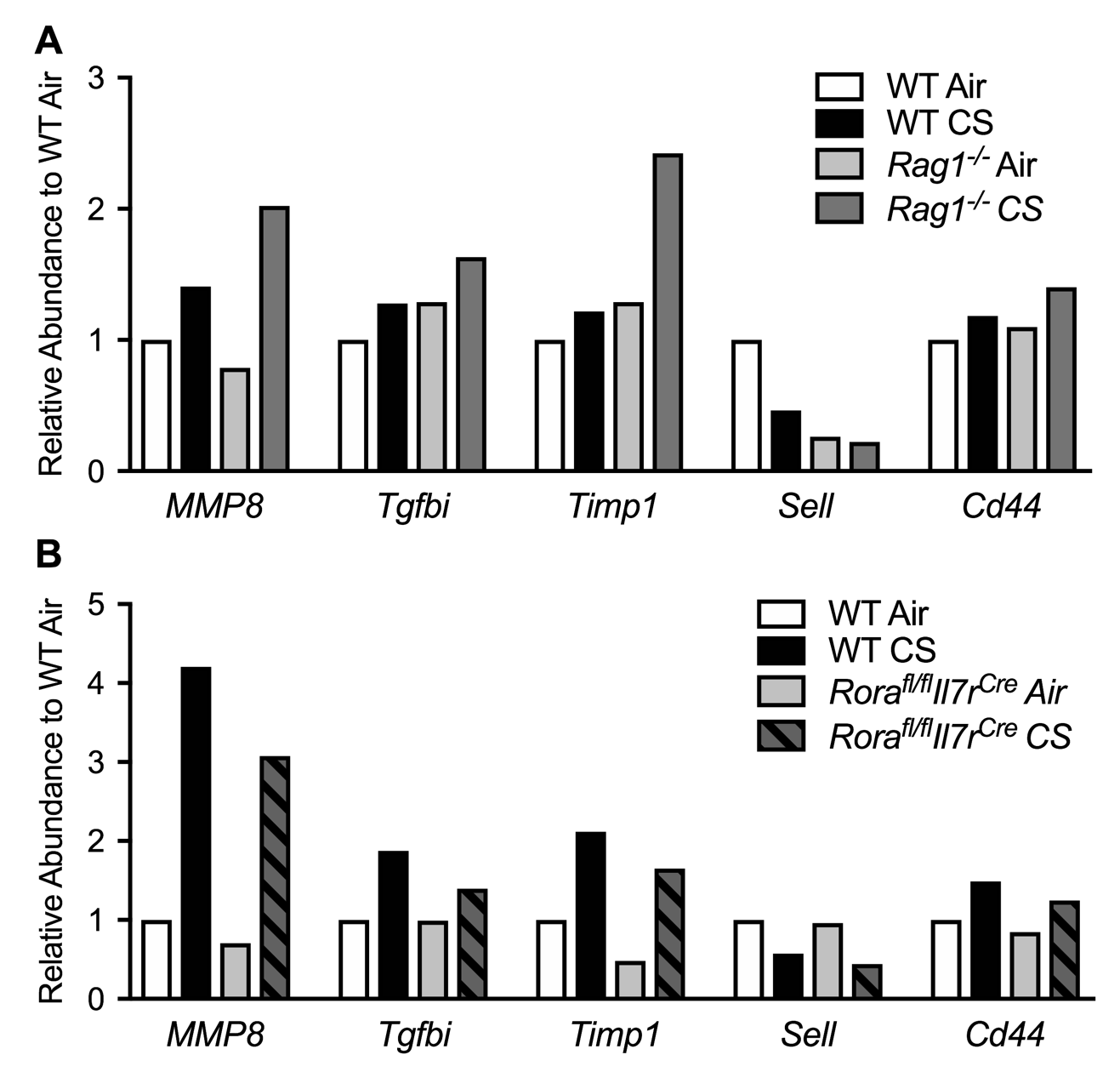

Supplement: Supplementary file 2 — Supplementary Fig. 2: T‐bet+ ILC1s and Rorγt+ ILC3s are increased and GATA3+ ILC2s are decreased in WT mice exposed to CS. Flow cytometric analysis of single cell suspensions from whole lungs of normal air‐ or CS‐exposed WT mice. (A) Flow cytometry gating of ILC1s, ILC2s and ILC3s. Quantification of (B) total ILCs, (C) ILC1s, (D) ILC2s, and (E) ILC3s. n = 5‐6 per group. Data are expressed as mean ± sem. * P < 0.05, ** P < 0.01. Panels: ILC1: Lin−CD90.2+CD45+IL‐7Rα+T‐bet+; ILC2: Lin−CD90.2+CD45+IL‐7Rα+GATA3+T‐bet−Rorγt−CD4−; ILC3: Lin−CD90.2+CD45+IL‐7Rα+Rorγt+CD4+/−. [file JLB-105-143-s002.tiff]
